# Transferrin Receptor Protein 1 Cooperates with mGluR2 To Mediate the Internalization of Rabies Virus and SARS-CoV-2

**DOI:** 10.1128/jvi.01611-22

**Published:** 2023-02-13

**Authors:** Xinxin Wang, Zhiyuan Wen, Huizhen Cao, Jie Luo, Lei Shuai, Chong Wang, Jinying Ge, Xijun Wang, Zhigao Bu, Jinliang Wang

**Affiliations:** a State Key Laboratory of Veterinary Biotechnology, Harbin Veterinary Research Institute, Chinese Academy of Agricultural Sciences, Harbin, People’s Republic of China; The Peter Doherty Institute for Infection and Immunity

**Keywords:** rabies virus, SARS-CoV-2, mGluR2, internalization, TfR1

## Abstract

Identification of bona fide functional receptors and elucidation of the mechanism of receptor-mediated virus entry are important to reveal targets for developing therapeutics against rabies virus (RABV) and severe acute respiratory syndrome coronavirus 2 (SARS-CoV-2). Our previous studies suggest that metabotropic glutamate receptor subtype 2 (mGluR2) functions as an entry receptor for RABV *in vitro*, and is an important internalization factor for SARS-CoV-2 *in vitro* and *in vivo*. Here, we demonstrate that mGluR2 facilitates RABV internalization *in vitro* and infection *in vivo*. We found that transferrin receptor 1 (TfR1) interacts with mGluR2 and internalizes with mGluR2 and RABV in the same clathrin-coated pit. Knockdown of TfR1 blocks agonist-triggered internalization of mGluR2. Importantly, TfR1 also interacts with the SARS-CoV-2 spike protein and is important for SARS-CoV-2 internalization. Our findings identify a novel axis (mGluR2-TfR1 axis) used by RABV and SARS-CoV-2 for entry, and reveal TfR1 as a potential target for therapeutics against RABV and SARS-CoV-2.

**IMPORTANCE** We previously found that metabotropic glutamate receptor subtype 2 (mGluR2) is an entry receptor for RABV *in vitro*, and an important internalization factor for SARS-CoV-2 *in vitro* and *in vivo*. However, whether mGluR2 is required for RABV infection *in vivo* was unknown. In addition, how mGluR2 mediates the internalization of RABV and SARS-CoV-2 needed to be resolved. Here, we found that mGluR2 gene knockout mice survived a lethal challenge with RABV. To our knowledge, mGluR2 is the first host factor to be definitively shown to play an important role in RABV street virus infection *in vivo*. We further found that transferrin receptor protein 1 (TfR1) directly interacts and cooperates with mGluR2 to regulate the endocytosis of RABV and SARS-CoV-2. Our study identifies a novel axis (mGluR2-TfR1 axis) used by RABV and SARS-CoV-2 for entry and opens a new door for the development of therapeutics against RABV and SARS-CoV-2.

## INTRODUCTION

Cell entry is the first step for a virus to establish an infection. Usually, viruses first bind to the cell surface by interacting with a specific receptor(s), and are then internalized through an inherent cellular internalization pathway (1, 2). Clathrin-mediated endocytosis (CME) (3), direct membrane fusion (4), macropinocytosis (5), and caveolin-mediated endocytosis (6) are the best-studied internalization pathways for virus infection (7). While the binding receptors used by different viruses vary, the internalization mechanisms are relatively conserved among different viruses (2, 8). Several cellular proteins have been reported to affect the entry of viruses. For example, the L-type calcium channel Ca_v_1.2 pore-forming subunit is an entry factor for severe acute respiratory syndrome coronavirus 2 (SARS-CoV-2) and influenza A virus (9, 10); GRP78 is an entry factor for Japanese encephalitis virus (11), and Coxsackievirus (12); and TIM-1 is an entry factor for Chikungunya virus and hepatitis C virus (13, 14). Identifying novel conserved entry factors and exploring the underlying mechanisms are essential to develop broad-spectrum antiviral drugs that block early infection.

Rabies virus (RABV) is the causative agent of rabies (15) and belongs to the genus *Lyssavirus* of the family *Rhabdviridae*. Humans are usually infected through contact with RABV-infected animals. About 60,000 rabies deaths are reported annually worldwide (15). Once the virus invades the central nervous system, no therapy has been proven to prevent death. RABV glycoprotein (G) is the only protein exposed on the surface of the viral envelope (16) and is responsible for receptor binding and viral entry (17, 18). After binding to the host receptor, RABV enters host cells through receptor-dependent CME (18–20).

SARS-CoV-2, the causative pathogen of coronavirus disease 2019 (COVID-19), belongs to the *Coronaviridae* family (21). As of October 15, 2022, the COVID-19 pandemic has resulted in more than 620 million confirmed cases and approximately 6.5 million deaths worldwide, according to the World Health Organization. The SARS-CoV-2 spike protein (S) is responsible for receptor binding and viral entry (21). After binding to specific cellular receptors, SARS-CoV-2 is internalized into cells through receptor-mediated CME or direct fusion at the plasma membrane (22, 23).

RABV and SARS-CoV-2 are completely different in their tissue tropism and consequent diseases after infection, however, both viruses can use metabotropic glutamate receptor subtype 2 (mGluR2) to facilitate cell entry (24, 25). Our previous studies suggested that mGluR2 functions an entry receptor for RABV *in vitro* (24), and an important internalization factor for SARS-CoV-2 *in vitro* and *in vivo* (25). However, whether mGluR2 facilitates RABV internalization *in vitro* and infection *in vivo* remains unknown. Moreover, how mGluR2 mediates the internalization of RABV and SARS-CoV-2 remains unclear.

In this study, we found that mGluR2 gene knockout mice survived a lethal challenge with RABV street strain. We further found that transferrin receptor protein 1 (TfR1) interacts with and cooperates with mGluR2 to mediate the internalization of RABV and SARS-CoV-2. Our study opens a new door for the development of therapeutics against RABV and SARS-CoV-2.

## RESULTS

### mGluR2 is important for RABV infection *in vivo*.

**FIG 1:**
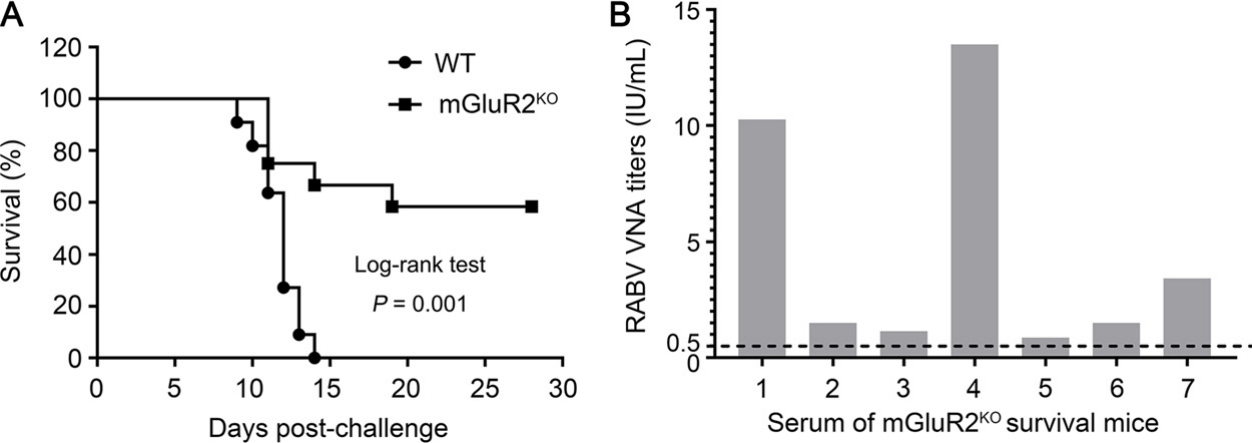
mGluR2 is important for RABV infection *in vivo*. (A) WT (*n* = 11) and mGluR2 gene knockout (mGluR2^KO^) (*n* = 12) mice were intramuscularly inoculated with 10 LD_50_ of street virus GX/09, and the survival rates of mice were monitoring for 4 weeks after infection. The data represent the sum of 2 independent experiments. (B) The titers of RABV VNA for the surviving mice were determined by using the WHO standard at 28-days postinfection.

We previously found that mGluR2 is a cellular receptor for RABV *in vitro* (24). We therefore investigated whether mGluR2 is important for RABV infection *in vivo* by using previously generated mGluR2 gene knockout (mGluR2^KO^) mice (25). Eleven wild-type (WT) mice and 12 mGluR2^KO^ mice were intramuscularly inoculated with RABV street strain GX/09 at a dose of 10 LD_50_ (50 percent lethal dose). In the 4 weeks after infection, all 11 WT mice died within 9 to 14 days of infection, whereas seven of the 12 mGluR2^KO^ mice survived (Fig. 1A and Table 1). For the mGluR2^KO^ mice, 4 died within 11 to 14 days of infection, and 1 died at 19 days postinfection. All of the dead mice displayed typical rabies disease signs, such as body tremors and hind limb paralysis (Table 1). Sera from the 7 surviving mGluR2^KO^ mice taken at 28 days postinfection were positive for RABV neutralization antibodies (Fig. 1B). These results strongly indicate that RABV uses mGluR2 as a cellular receptor to infect *in vivo*.

**TABLE 1:** Neurologic symptoms and survival of mice inoculated with RABV street strain GX/09

Days	WT mice			mGluR2^KO^ mice		
	Neural symptom	Dead	Survival	Neural symptom	Dead	Survival
1	0	0	11	0	0	12
2	0	0	11	0	0	12
3	0	0	11	0	0	12
4	0	0	11	0	0	12
5	0	0	11	0	0	12
6	0	0	11	0	0	12
7	0	0	11	0	0	12
8	5	0	11	2	0	12
9	10	1	10	2	0	12
10	9	1	9	3	0	12
11	7	2	7	1	3	9
12	3	4	3	1	0	9
13	1	2	1	1	0	9
14	0	1	0	0	1	8
15	0	0	0	0	0	8
16	0	0	0	0	0	8
17	0	0	0	0	0	8
18	0	0	0	1	0	8
19	0	0	0	0	1	7
20	0	0	0	0	0	7
21	0	0	0	0	0	7
22	0	0	0	0	0	7
23	0	0	0	0	0	7
24	0	0	0	0	0	7
25	0	0	0	0	0	7
26	0	0	0	0	0	7
27	0	0	0	0	0	7
28	0	0	0	0	0	7

### mGluR2 is required for the endocytosis of RABV.

**FIG 2:**
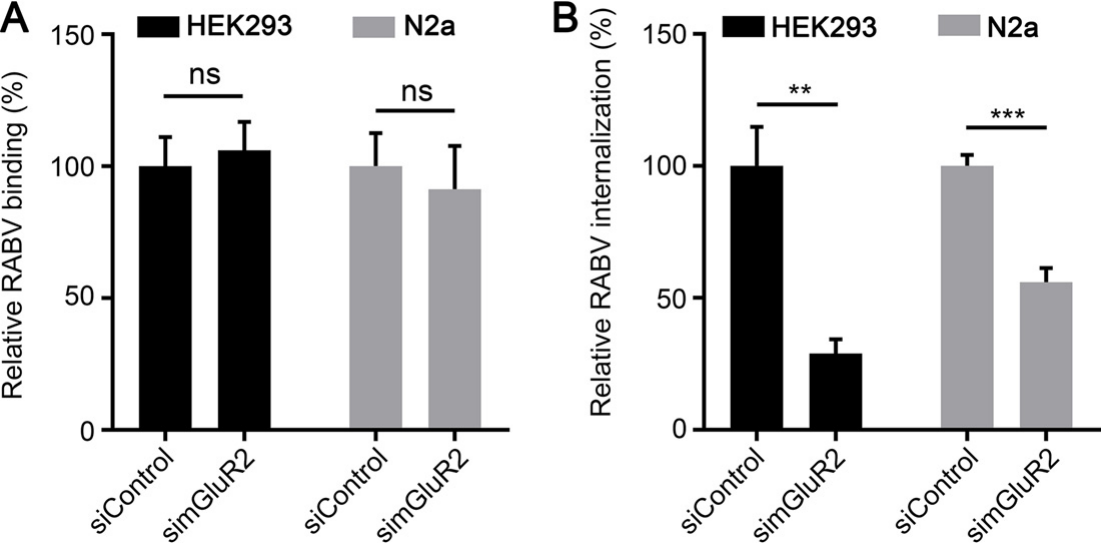
mGluR2 is required for RABV internalization. (A and B) RABV binding (A) and internalization (B) assays were performed in mGluR2-silenced HEK293 cells and N2a cells. Viral binding or internalization was quantified by normalization to the respective control cells. The data shown are the means ± SDs of 3 independent experiments or replicates. The two-tailed unpaired Student's *t* test was used for the statistical analysis. ns, not significant, **P < 0.01; ***P < 0.001.

Given that mGluR2 is important for RABV infection *in vitro* and *in vivo*, we next asked how mGluR2 influences RABV entry. First, we examined which stage of RABV entry involves mGluR2. RNA interference (RNAi) assays were performed to determine whether mGluR2 affects the cell binding or internalization of RABV as previously described (26). Briefly, mGluR2-silenced HEK293 cells, N2a cells (a mouse neuroblastoma cell line), and control cells were incubated with a recombinant RABV ERA strain that expresses enhanced green florescence protein (ERA-EGFP), at 4°C for 1 h and then washed to remove unbound virus. Then, the cells were shifted to 37°C for 2.5 h to allow the internalization of bound viruses. After being washed with phosphate buffered saline (PBS) or trypsin to remove the RABV particles bound to cell surface, the cells were lysed for quantitative real-time PCR (qPCR) to detect RABV binding to or entry into cells. We found that mGluR2 silencing had no effect on RABV binding to HEK293 cells or N2a cells (Fig. 2A), whereas the internalization of RABV was significantly decreased in both cell types with mGluR2 silencing (Fig. 2B). These results indicate that mGluR2 is important for the internalization of RABV.

### TfR1 interacts with mGluR2.

**FIG 3:**
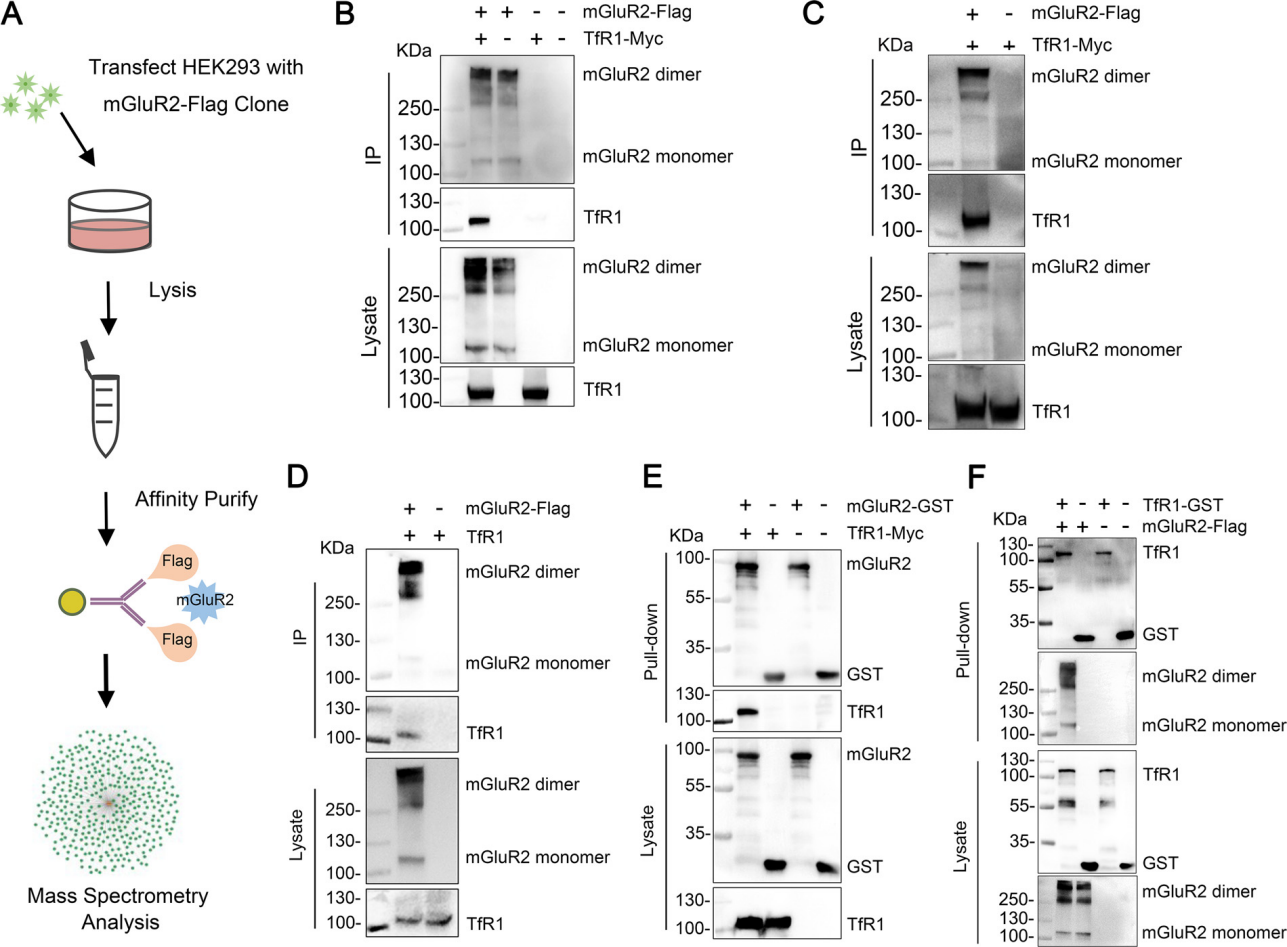
TfR1 interacts with mGluR2. (A) Schematic representation of the AP-MS approach used to identify mGluR2-protein interactions in HEK293 cells. (B and C) HEK293 cells were co-transfected with mGluR2-Flag and TfR1-Myc, then cells were solubilized by NP-40 lysis buffer (B) or RIPA lysis buffer (C), and then subjected to immunoprecipitation (IP) by using anti-Flag agarose beads. Representative Western blots of whole cell lysates and eluates after IP are shown. (D) Co-immunoprecipitation of mGluR2-Flag with endogenous TfR1 in HEK293 cells. (E and F) Purified mGluR2-GST protein (E) or TfR1-GST protein (F) was pooled with the lysate from TfR1-Myc- (E) or mGluR2-Flag- (F) transfected HEK293 cells and then pulled down by using anti-GST beads. The GST protein was used as the negative control.

The mGluR2 belongs to the G-protein-coupled receptor (GPCR) family (27). The best-characterized pathway for GPCR endocytosis occurs through clathrin- and dynamin-dependent processes (28). However, activated GPCRs do not initiate clathrin-coated pit (CCP) formation *de novo* and are recruited into preexisting CCPs (29, 30). It is highly likely, therefore, that other host factors capable of initiating endocytosis *de novo* are involved in the endocytosis of RABV. To understand the role of mGluR2 in RABV infection, we performed an affinity purification coupled to mass spectrometry (AP-MS) study to obtain a high-quality mGluR2 interactome, screened for host factors that interact with mGluR2, and investigated their function during RABV infection (Fig. 3A). We analyzed 3 independent replicates for mGluR2 interactors, and only a protein for which the number of unique peptides was at least twice that for the corresponding control in at least 2 independent replicates was identified as a specific interactor. Using these criteria, a total of 363 proteins were found to interact with the mGluR2-Flag (Table S1).

Among the mGluR2-binding proteins identified, we found that transferrin receptor 1 (TfR1) protein was a strong candidate interactor. TfR1 is a typical membrane-bound protein and undergoes CME *de novo* (31). GPCR can enter TfR1-containing CCPs, and GPCR- and TfR1- containing CCPs have longer cell surface residence time than that of only TfR1-containing CCPs (32). Interestingly, the lifetime of RABV endocytosis is also longer than that of classic TfR1 endocytosis (19, 33, 34). More importantly, in a recent study, we demonstrate that TfR1 is an entry factor for RABV (26). Therefore, we performed a co-immunoprecipitation assay using Flag-tagged mGluR2 protein (mGluR2-Flag) and Myc-tagged TfR1 protein (TfR1-Myc) in plasmid-transfected HEK293 cells to validate the interaction. We found that mGluR2 and TfR1 interacted specifically (Fig. 3B and C). We then performed a co-immunoprecipitation assay using mGluR2-Flag in plasmid-transfected HEK293 cells to determine whether mGluR2 interacts with endogenous TfR1, and found that endogenous TfR1 co-precipitated only in the presence of mGluR2 (Fig. 3D). We also performed pulldown assays to determine whether mGluR2 interacts with TfR1 directly. The GST-tagged mGluR2 ectodomain (mGluR2-GST) and TfR1 ectodomain (TfR1-GST) were purified, respectively, then mGluR2-GST or TfR1-GST were pooled with the lysate of TfR1-Myc or mGluR2-Flag plasmid-transfected HEK293 cells for pulldown assays. The results showed that the ectodomain of mGluR2 and the ectodomain of TfR1 interact (Fig. 3E and F). These results reveal that TfR1 interacts with mGluR2.

### mGluR2, RABV, and TfR1 internalize together.

**FIG 4:**
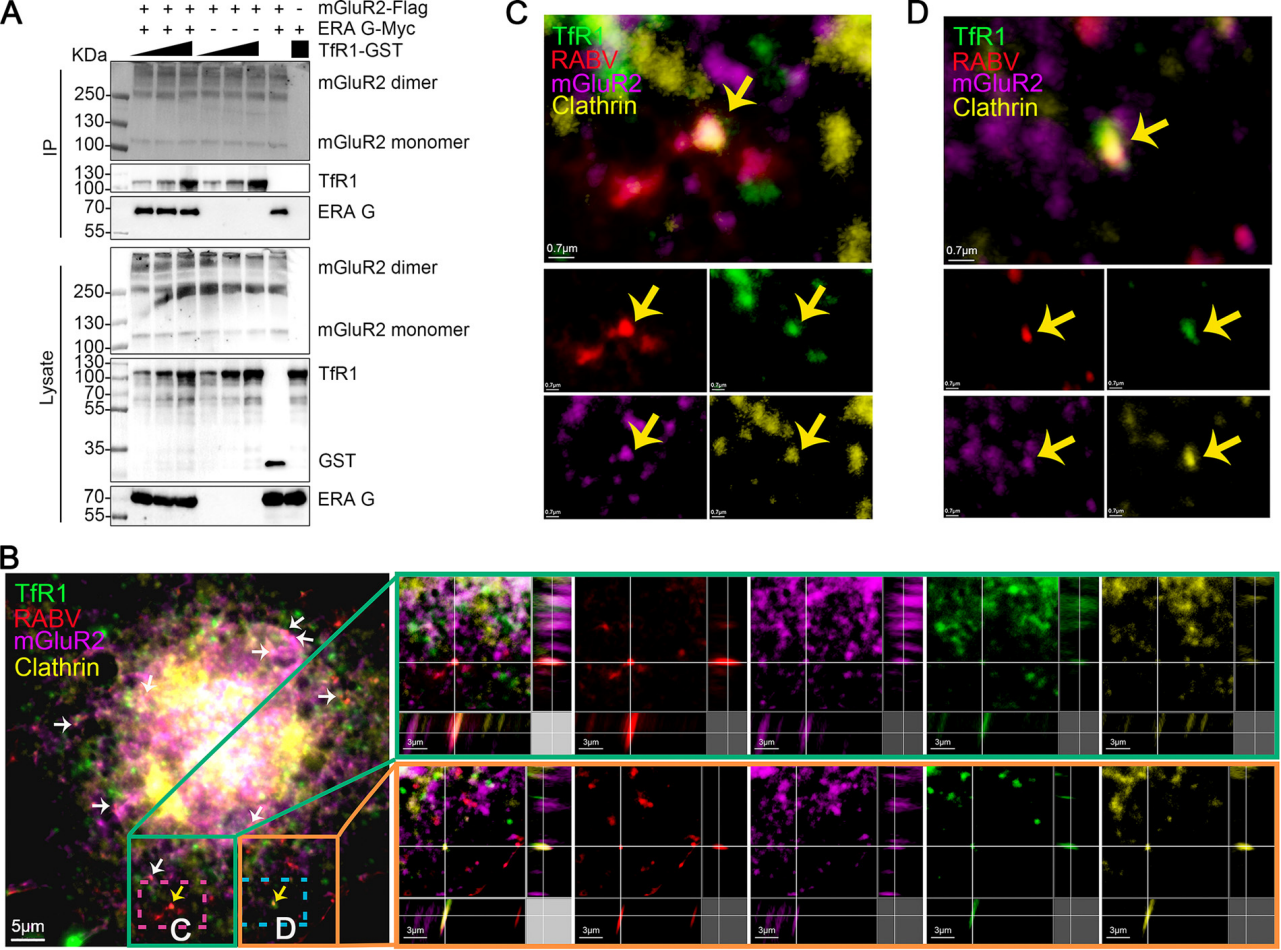
mGluR2, RABV, and TfR1 internalize together. (A) Increasing amounts of TfR1-GST were respectively mixed with mGluR2-Flag-conjugated beads, then the lysate from RABV G-Myc-transfected HEK293 cells was added for use in the protein interaction competition assay. (B to D) N2a cells were infected with ERA-N-mCherry for 10 min at 37°C, and then multiplex immunofluorescence was performed. The arrows indicate the colocalization of TfR1 (green), ERA-N-mCherry (red), clathrin (yellow), and mGluR2 (purple). Two representative co-localizations (scale bar, 3 μm) (yellow arrows) are shown in three dimensions (B); the dashed box is magnified at the indicated location of the same image (scale bar, 0.7 μm) (C and D).

Our previous studies (26) combined with the above results demonstrate that TfR1, mGluR2, and RABV G interact with each other, which suggests that they might form a complex. To test this hypothesis, we performed a protein interaction competition assay. Increasing amounts of TfR1-GST were respectively mixed with mGluR2-Flag-conjugated beads, then the lysate of cells expressing ERA G-Myc was added. The result showed that different concentrations of TfR1-GST did not disrupt the interaction between mGluR2-Flag and ERA G-Myc (Fig. 4A), which indicates that mGluR2, RABV G, and TfR1 have the potential to form a complex.

We next tested whether the complex of mGluR2, RABV, and TfR1 is internalized together upon RABV infection. We used multiplex immunofluorescence with TSA staining to detect ERA-N-mCherry, a recombinant ERA expressing an additional open reading frame of the N protein fused with mCherry, TfR1, mGluR2 and clathrin in ERA-N-mCherry-infected N2a cells. We observed clear co-localization of TfR1, ERA-N-mCherry, mGluR2, and clathrin (Fig. 4B to D), thereby demonstrating that RABV, TfR1, and mGluR2 internalize together within the same CCP.

### TfR1 and TTP are required for agonist-triggered endocytosis of mGluR2.

**FIG 5:**
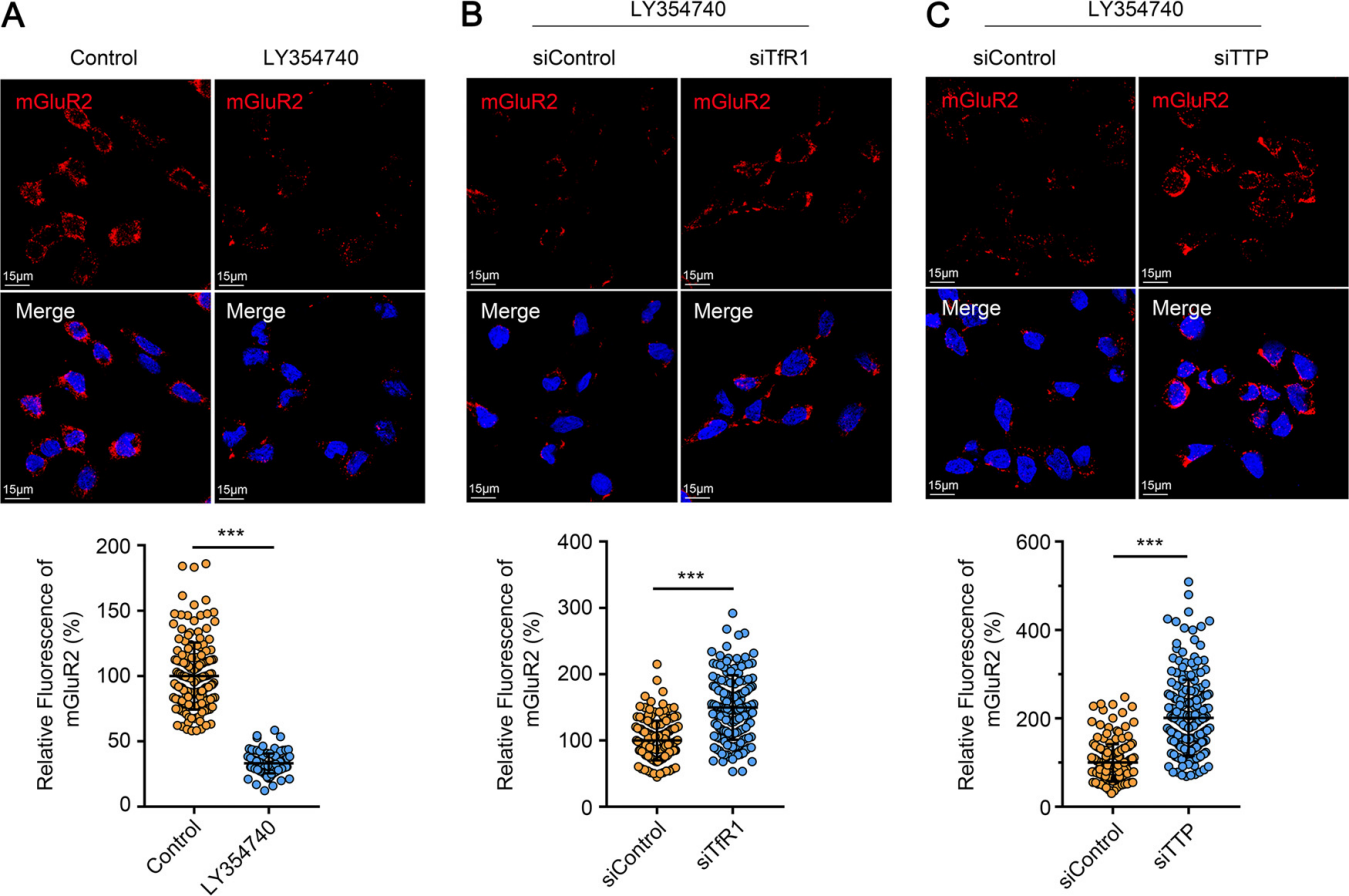
TfR1 and TTP are required for agonist-triggered mGluR2 endocytosis. (A) HEK293 cells were treated with or without LY354740 for 30 min at 37°C, and then were stained with an antibody against mGluR2 (red). Cell nuclei were stained with Hoechst 33342. Representative images are shown. The fluorescence signal of mGluR2 was quantified by using ZEN software. The relative fluorescence of mGluR2 was quantified by normalization to control cells. The circles represent individual datapoints. At least 100 cells per sample were quantified. (B and C) TfR1-silenced (B) or TTP-silenced (C) HEK293 cells were treated with LY354740 as described in (A). Representative images are shown. The data represent the sum of three independent experiments. The two-tailed unpaired Student's *t* test was used for the statistical analysis. ***P < 0.001.

Until now, the details of the endocytosis of mGluR2 have remained unclear. Since TfR1 interacts and co-localizes with mGluR2 in the same CCP, we asked whether TfR1 is involved in the endocytosis of mGluR2. LY354740 is a potent and highly selective agonist of mGluR2 (35). We therefore tested whether LY354740 triggers the endocytosis of mGluR2 by using our microscopy-based assay. The cell surface fluorescence intensity of mGluR2 in LY354740-treated cells was significantly decreased compared with that of mock-treated cells (Fig. 5A). This result indicates that LY354740 could trigger the endocytosis of mGluR2.

Next, we tested whether TfR1 is required for agonist-triggered endocytosis of mGluR2. Upon LY354740 stimulation, the cell surface fluorescence intensity of mGluR2 was higher in TfR1-silenced HEK293 cells than in control cells (Fig. 5B), which indicates that TfR1 is required for agonist-triggered endocytosis of mGluR2. Of note, we also found that the cell surface fluorescence intensity of mGluR2 in TTP-silenced HEK293 cells was significantly increased compared with that of control cells (Fig. 5C). These results indicate that endocytosis of TfR1 is required for agonist-triggered endocytosis of mGluR2 in HEK293 cells.

### TfR1 is important for the internalization of SARS-CoV-2.

**FIG 6:**
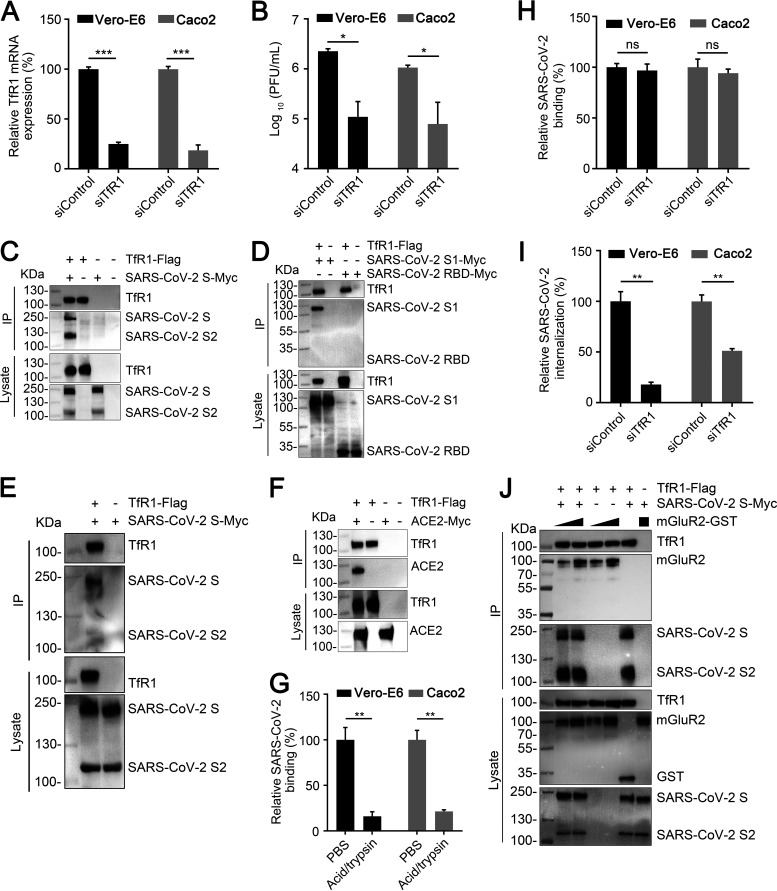
TfR1 is important for the internalization of SARS-CoV-2. (A) The TfR1 mRNA level in the indicated siRNA-transfected Vero-E6 cells or Caco2 cells was measured by qPCR. (B) TfR1-silenced Vero-E6 cells or Caco2 cells were infected with SARS-CoV-2 HRB25 strain. At 24 h postinfection, virus in the culture supernatant was detected by use of plaque assays. (C and D) Co-immunoprecipitation of TfR1-Flag and SARS-CoV-2 S-Myc (C), SARS-CoV-2 S1-Myc, or SARS-CoV-2 RBD-Myc (D) in plasmid-transfected HEK293 cells. (E) Co-immunoprecipitation of TfR1-Flag and SARS-CoV-2 S-Myc in plasmid-transfected Vero-E6 cells. (F) Co-immunoprecipitation of TfR1-Flag and ACE2-Myc in plasmid-transfected HEK293 cells. (G) Cells were incubated with SARS-CoV-2 for 1 h at 4°C, then washed with acid buffer/trypsin, and lysed for qPCR to detect virus bound to the cell surface. (H and I) SARS-CoV-2 binding (H) and internalization (I) assays were performed in TfR1-silenced Vero-E6 cells or Caco2 cells. (J) Increasing amounts of mGluR2-GST were respectively mixed with TfR1-Flag-conjugated beads, and then with the lysate from SARS-CoV-2 S-Myc-transfected HEK293 cells for the protein interaction competition assay. In panels (A), (B), and (G to I), error bars represent the means ± SDs of 3 independent experiments or replicates. The two-tailed unpaired Student's *t* test was used for the statistical analysis. ns, not significant, *P < 0.05; **P < 0.01; ***P < 0.001.

Our recent study found that mGluR2 is also an internalization factor for SARS-CoV-2 (25). We therefore speculated that TfR1 could also affect the internalization of SARS-CoV-2. We first determined whether TfR1 is required for SARS-CoV-2 infection by using RNAi assays in Vero-E6 cells and Caco2 cells. qPCR analysis confirmed that TfR1 mRNA expression was significantly reduced in Vero-E6 cells and Caco2 cells at 24 h posttransfection (Fig. 6A). TfR1-silenced cells were infected with a SARS-CoV-2 human isolate (HRB25) and the infectious titers in the supernatants of the infected cells were detected by performing plaque assays at 24 h postinfection. We found that knockdown of TfR1 significantly decreased the viral titers in the supernatant (Fig. 6B), indicating that TfR1 is required for SARS-CoV-2 infection. The SARS-CoV-2 S protein is responsible for receptor binding and viral entry (36). The S protein comprises the S1 and S2 subdomains. The S1 subdomain contains the receptor binding domain (RBD) and is responsible for binding to specific receptors. The S2 subdomain contains a fusion peptide and is responsible for the fusion of the viral membrane with the cellular membrane (36, 37). We therefore investigated whether the SARS-CoV-2 S protein interacts with TfR1 in HEK293 cells by using co-immunoprecipitation assays. We found that TfR1 interacted well with full-length SARS-CoV-2 S (Fig. 6C) and the S1 subdomain, but poorly with the RBD (Fig. 6D). Interestingly, the S2 subdomain was co-precipitated in the presence of TfR1 (Fig. 6C). These results demonstrate that TfR1 interacts with SARS-CoV-2 S. We further tested whether SARS-CoV-2 S interacts with TfR1 in Vero-E6 cells by using co-immunoprecipitation assays and found that TfR1 interacted with SARS-CoV-2 S (Fig. 6E). Of note, we also found that TfR1 interacted with ACE2 (Fig. 6F), a binding receptor of SARS-CoV-2 (22).

We then examined the internalization of SARS-CoV-2 in TfR1-silenced Vero-E6 cells and Caco2 cells by qPCR. The cell surface-bound virus could be efficiently removed by acid buffer/trypsin (Fig. 6G). Compared with siControl-transfected cells, TfR1 silencing effectively decreased the internalization of SARS-CoV-2 but did not affect SARS-CoV-2 binding (Fig. 6H and I). These results demonstrate that TfR1 is an internalization factor for SARS-CoV-2.

Given that mGluR2 is also an internalization factor of SARS-CoV-2 (25), we next examined whether SARS-CoV-2 S forms a complex with TfR1 and mGluR2 by using the protein interaction competition assay. Increasing amounts of mGluR2-GST were respectively mixed with TfR1-Flag-conjugated anti-Flag beads, then the lysate of cells expressing SARS-CoV-2 S-Myc was added. The result showed that different concentrations of mGluR2-GST did not disrupt the interaction between TfR1-Flag and SARS-CoV-2 S-Myc (Fig. 6J), which indicates that TfR1, mGluR2, and SARS-CoV-2 S have the potential to form a complex.

### TfR1 is important for SARS-CoV S- and MERS-CoV S-mediated internalization.

**FIG 7:**
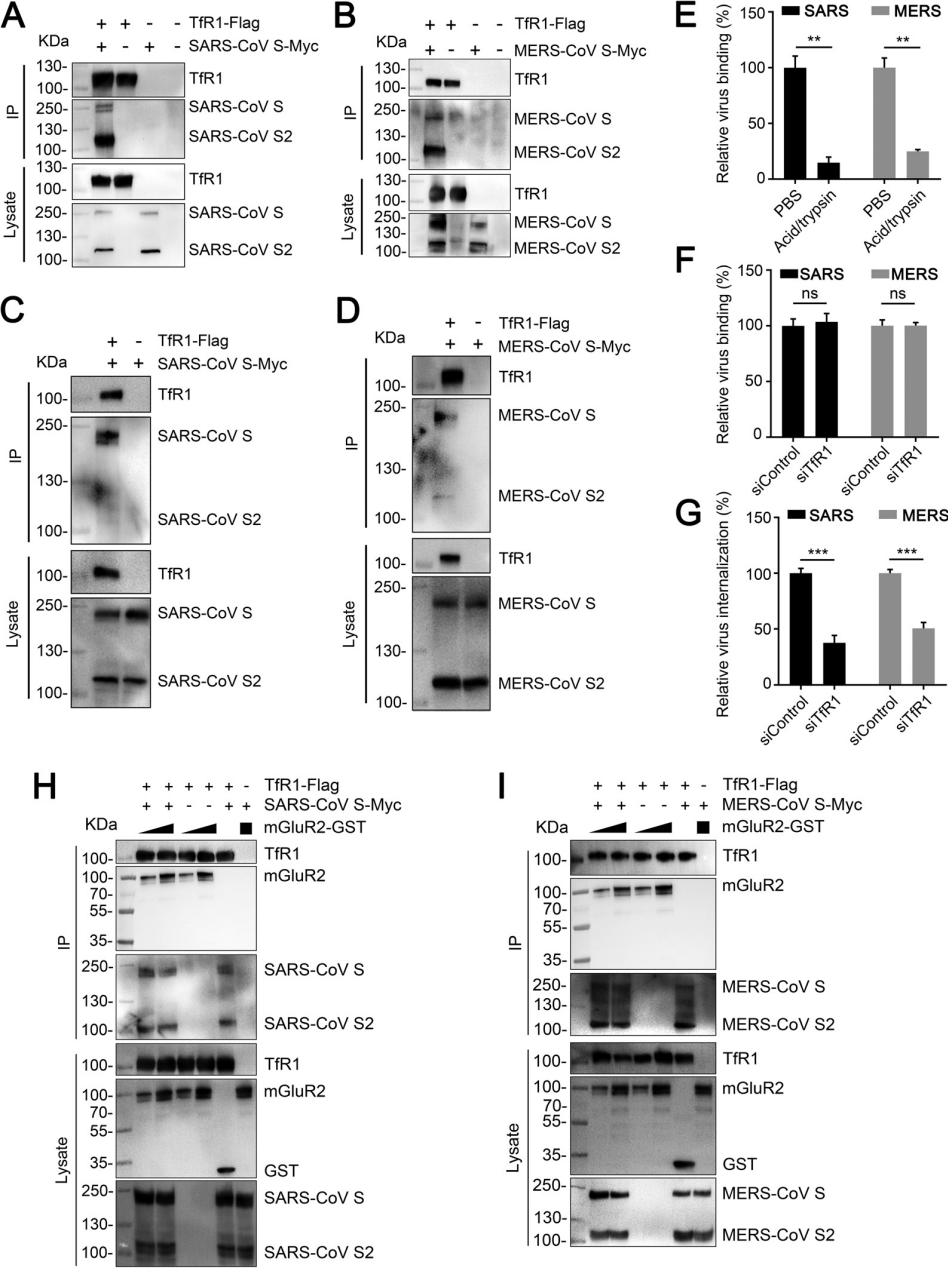
TfR1 is important for SARS-CoV S- and MERS-CoV S-mediated internalization. (A and B) Co-immunoprecipitation of TfR1-Flag and SARS-CoV S-Myc (A), or MERS-CoV S-Myc (B) in plasmid-transfected HEK293 cells. (C and D) Co-immunoprecipitation of TfR1-Flag and SARS-CoV S-Myc (C), or MERS-CoV S-Myc (D) in plasmid-transfected Vero-E6 cells. (E) Cells were incubated with rVSV-SARS-CoV-S, or rVSV-MERS-CoV-S for 1 h at 4°C, then washed with acid buffer/trypsin, and lysed for qPCR to detect virus bound to the cell surface. (F and G) rVSV-SARS-CoV-S, or rVSV-MERS-CoV-S binding (F) and internalization (G) assays were performed in TfR1-silenced Vero-E6 cells. (H and I) Increasing amounts of mGluR2-GST were respectively mixed with TfR1-Flag-conjugated beads, and then with the lysate from SARS-CoV S-Myc- (H) or MERS-CoV S-Myc- (I) transfected HEK293 cells for the protein interaction competition assay. In panels (E to G), error bars represent the means ± SDs of 3 independent experiments or replicates. The two-tailed unpaired Student's *t* test was used for the statistical analysis. ns, not significant, **P < 0.01; ***P < 0.001.

Severe acute respiratory syndrome coronavirus (SARS-CoV) and Middle East respiratory syndrome coronavirus (MERS-CoV) are also highly pathogenic betacoronaviruses (36). We therefore asked whether TfR1 is also important for the internalization of SARS-CoV and MERS-CoV. Results from co-immunoprecipitation assays showed that TfR1 interacted with SARS-CoV S and MERS-CoV S in HEK293 cells (Fig. 7A and B) and Vero-E6 cells (Fig. 7C and D). We then examined whether TfR1 affects the internalization of SARS-CoV and MERS-CoV by using qPCR. Recombinant vesicular stomatitis viruses (VSV) expressing SARS-CoV S (rVSV-SARS-CoV-S) or MERS-CoV S (rVSV-MERS-CoV-S) were used to substitute for authentic SARS-CoV and MERS-CoV, respectively, as described previously (38). The results showed that acid buffer/trypsin could efficiently remove the surface-bound rVSV-SARS-CoV-S and rVSV-MERS-CoV-S virus on Vero-E6 cells (Fig. 7E), and that knockdown of TfR1 significantly decreased the internalization of both viruses, but had no effect on binding to the cells (Fig. 7F and G), indicating that TfR1 is also important for the internalization of SARS-CoV and MERS-CoV. We then examined whether SARS-CoV S or MERS-CoV S forms a complex with TfR1 and mGluR2 by using the protein interaction competition assay. We found that different concentrations of mGluR2-GST did not disrupt the interaction between TfR1-Flag and SARS-CoV S-Myc or MERS-CoV S-Myc (Fig. 7H and I), which indicates that TfR1, mGluR2, and SARS-CoV S or MERS-CoV S have the potential to form a complex.

## DISCUSSION

Previous studies have demonstrated that RABV may use multiple receptors to enter cells (39). Several host factors, including nAchR (40), NCAM (41), p75NTR (42), and mGluR2 (24), have been proposed as potential receptors *in vitro*. However, no direct evidence indicates nAchR is important for RABV infection *in vivo*; NCAM-deficient mice show delayed death from rabies (41) and rabies-infected knockout mice that lack all the extracellular receptor domains of p75NTR do not change the mortality of rabies (43). In this study, we demonstrated that mGluR2 is a functional entry receptor for RABV *in vivo*. To our knowledge, mGluR2 is the first host factor to be definitively demonstrated to play an important role in RABV street virus infection *in vivo* (39). It is interesting that about 45% of mGluR2^KO^ mice died as a result of their RABV infection, suggesting that mGluR2 is an important but not the only receptor for RABV infection. Our findings further suggest that RABV may use multiple receptors to enter cells.

The best-characterized pathway for GPCR endocytosis occurs through CME (28). While the mechanism of mGluR2 endocytosis remains unknown, our results indicate that mGluR2 is likely internalized via TfR1-dependent CME. Previous studies have revealed that 3 populations of CCPs exist at the plasma membrane of HEK293 cells after stimulation by the ligand of the β2-adrenergic receptor (β2AR), a member of the GPCR family: about 25% of the CCPs contained only β2AR, 50% contained both β2AR and TfR1, and 25% contained only TfR1 (44). The β2AR clustering prolongs the half-lifetime of CCPs (32); therefore, the endocytosis time for TfR1 in β2AR-containing CCPs should be longer than that in CCPs without β2AR. The endocytosis of TfR1 in mGluR2-containing CCPs may also take longer, which may explain why RABV endocytosis takes longer than classic CME. In this study, we found that TfR1 is required for the endocytosis of SARS-CoV-2, SARS-CoV, and MERS-CoV. It is interesting that TfR1 is involved in cell entry for many pathogens, including Machupo virus (45), Canine parvovirus (46), hepatitis C virus (47), and Plasmodium vivax (48). The mechanistic details of the CME of different viruses involving TfR1-containing CCPs warrant further study.

Most GPCRs are transported to an endocytic pathway after internalization (49, 50). In our previous studies, we found that mGluR2 and TfR1 co-localize with RABV in early and late endosomes, respectively, and the interaction between mGluR2 or TfR1 and RABV G is not affected by acidic conditions (24) (26). These results indicate that mGluR2 likely cooperates with TfR1 to regulate RABV endosomal transport. A previous study showed that heteromerization of interclass GPCR affects the localization and trafficking of mGluR2 (51). The presence of serotonin 5-HT_2A_ receptor (5-HT_2A_R) in cells stably expressing mGluR2 increased mGluR2 co-localization with the early endosome marker Rab5, the late endosome marker Rab7, and TfR1, respectively (51). Whether 5-HT_2A_R or other GPCRs are involved in mGluR2- and TfR1-mediated RABV endosomal transport deserves further study.

SARS-CoV-2 is internalized into cells via direct fusion at the plasma membrane in the presence of TMPRSS2 or via receptor-dependent CME, followed by fusion at the endosome (22, 23, 52). Different SARS-CoV-2 variants might use different pathways to enter cells (53, 54). Our study found that TfR1 is required for SARS-CoV-2 internalization into Vero-E6 cells. Given that SARS-CoV-2 mainly enters Vero-E6 cells through the endosomal route (22), TfR1 is likely required by the SARS-CoV-2 variants that preferentially enter cells via the endosomal route, such as Omicron BA.1 (53). We also found that TfR1 interacts with ACE2, a well-known binding receptor of SARS-CoV-2 (22). This result indicates that TfR1 is a downstream effector of ACE2 and cooperates with ACE2 to mediate SARS-CoV-2 endocytosis. Several studies have suggested that there are other receptors for SARS-CoV-2 infection besides ACE2, such as AXL, KREMEN1, and ASGR1 (55, 56). Whether TfR1 plays a role in SARS-CoV-2 infection via other potential receptors remains to be explored.

**FIG 8:**
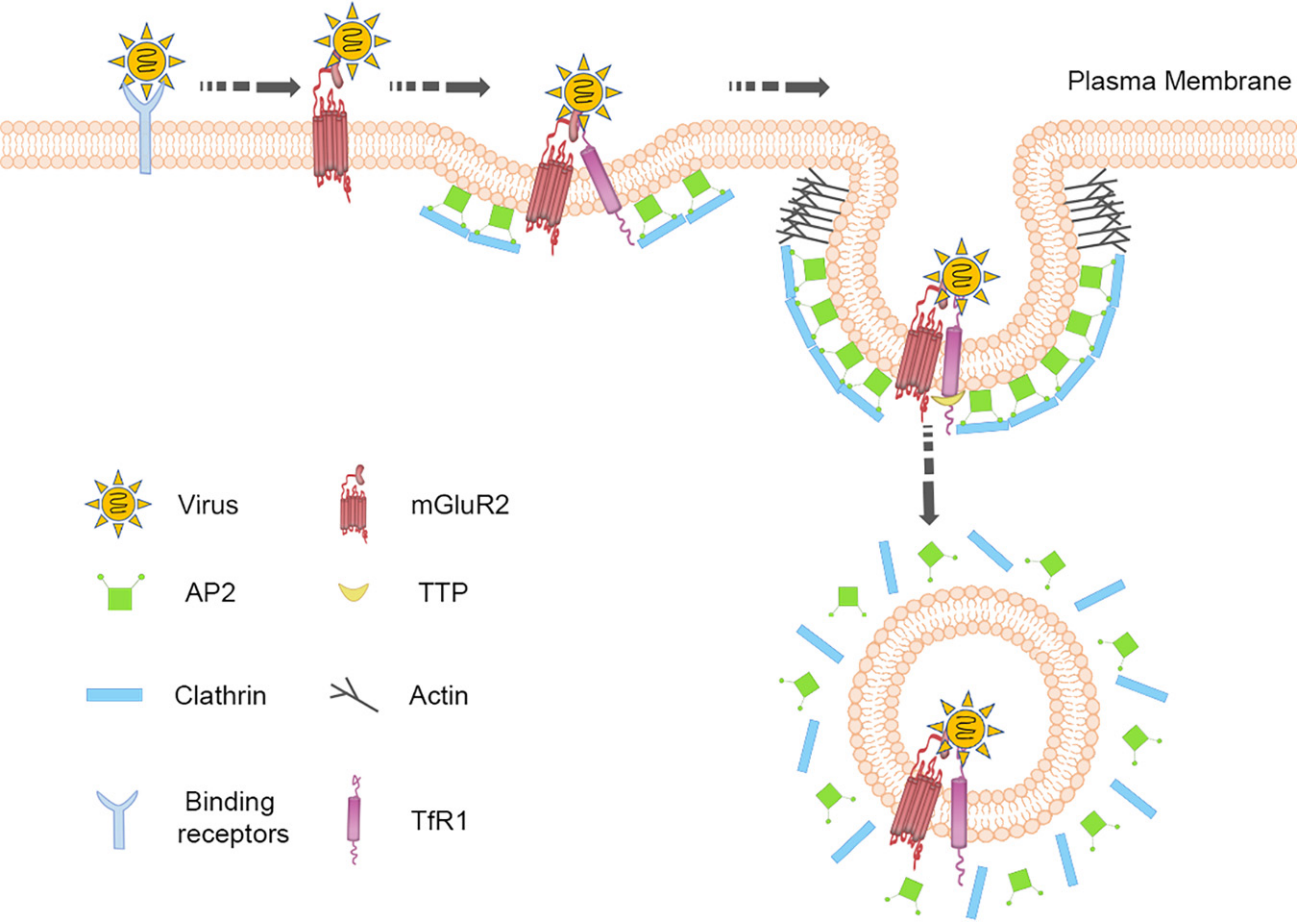
Proposed model for the role of mGluR2 and TfR1 in RABV or SARS-CoV-2 entry. The virus first binds to a specific receptor(s) at the cell surface, then the virus interacts with and activates mGluR2. The virus-mGluR2 complex migrates to a TfR1-containing pre-existing CCP, where it interacts with TfR1 and hijacks the endocytic signaling of TfR1 to enter the cell.

It seems likely that TfR1 and mGluR2 play a conservative role in viral internalization. We, therefore, propose the following model for the internalization of RABV and SARS-CoV-2 (Fig. 8). First, the virus binds to a specific receptor(s) at the cell surface. This receptor, for example, ACE2, is activated by the virus and then the virus-receptor complex interacts with and activates mGluR2. The virus-mGluR2 complex migrates to a TfR1-containing preexisting CCP, where it interacts with the TfR1 and hijacks the endocytic signaling of TfR1 to enter cells. Targeting TfR1 may present a novel approach for the development of pan-antiviral therapeutic interventions.

## MATERIALS AND METHODS

### Cell lines.

HEK293 cells (ATCC, CRL-1573), N2a cells (ATCC, CCL-131), Vero-E6 cells (ATCC, CRL-1586), and Caco2 cells (ATCC, HTB-37) were maintained in Dulbecco’s modified Eagle’s medium (DMEM) supplemented with 10% fetal bovine serum (FBS), 1% penicillin/streptomycin, and l-glutamine in 5% CO_2_.

### Viruses.

ERA strain was maintained in our laboratory. Recombinant ERA expressing EGFP (ERA-EGFP), recombinant ERA expressing mCherry, in which the ERA-N and mCherry in-frame fusion gene was inserted between the ERA P and M genes as an additional transcription unit (ERA-N-mCherry), was generated as described previously (57, 58). Rabies street virus strain GX/09 (59) was passaged in mouse brain and titrated to determine the intramuscular 50% lethal dose (LD_50_) in mice before the challenge study. VSV chimeras expressing the S protein of SARS-CoV (rVSV-SARS-CoV-S) or MERS-CoV (rVSV-MERS-CoV-S) were generated as previously described (38). SARS-CoV-2/HRB25/human/2020/CHN (HRB25, GISAID access no. EPI_ISL_467430) was maintained in our laboratory. All experiments with infectious SARS-CoV-2 were performed in the biosafety level 4 facilities at the Harbin Veterinary Research Institute (HVRI) of the Chinese Academy of Agricultural Sciences (CAAS).

### Ethics statement.

All experiments with RABV GX/09 virus were conducted in the animal biosafety level 3 (ABSL3) facility in the HVRI, CAAS, which is approved for such use by the Ministry of Agriculture and Rural Affairs of China. The protocol for the animal studies was approved by the Committee on the Ethics of Animal Experiments of the HVRI, CAAS (approval number 190212-01).

### Animal experiments.

All 6 to 8-week-old C57BL/6J mice were purchased from Beijing Vital River Laboratory Animal Technology. mGluR2^KO^ mice were generated by crossing and using the CRISPR/Cas9 system (25). WT and mGluR2^KO^ mice were intramuscularly inoculated with 10 LD_50_ of RABV GX/09 in a volume of 100 μL. All mice were observed for 28 days for signs of sickness and death. Survival rates and statistical significance were determined by using GraphPad Prism software.

### Plasmids.

The generation of mGluR2-Flag, ACE2-Myc, SARS-CoV-2 S-Myc, SARS-CoV-2 S1-Myc, SARS-CoV-2 RBD-Myc, SARS-CoV S-Myc, and MERS-CoV S-Myc was described previously (24, 25). Human TfR1 cDNA was cloned into the pCAGGS-Flag or pCAGGS-Myc vector as indicated in our study, and confirmed by sequencing analysis.

### Neutralization assay.

RABV virus neutralizing antibody (VNA) levels in serum samples were detected by using a fluorescent antibody virus neutralization test (60). The titers of RABV VNA were expressed in international units (IU)/mL by comparison with a serum standard that was equivalent to 0.5 IU/mL of VNA based on the WHO standard as a reference.

### RNAi.

The RNAi assay was performed as previously described (25). Briefly, siRNA targeting mGluR2 (Ambion, s6195), TfR1 (human, Ambion, s727), TTP (Ambion, s528796), or non-targeting siRNA, was mixed with Opti-MEM medium containing 1 μL of Lipofectamine RNAiMAX transfection reagent in a volume of 120 μL per well on 24-well plates. After a 30 min incubation at room temperature, cells were seeded into siRNA-coated 24-well plates in a volume of 500 μL per well. At 24 h after the siRNA transfection, TfR1 mRNA was assessed by use of qPCR. At 60 h posttransfection, the cells were infected with HRB25 (MOI = 0.01 for Vero-E6 cells; MOI = 0.05 for Caco2 cells) for further studies. The siRNA sequences targeting green monkey TfR1 were as follows: siTfR1-green monkey, Sense: 5′-GUCAAAGACAGUGCUCAAAdTdT-3′, Antisense: 5′-UUUGAGCACUGUCUUUGACdTdT-3′.

### Viral infection assay.

The siTfR1-silenced Vero-E6 cells or Caco2 cells were infected with HRB25 (MOI = 0.01 for Vero-E6 cells; MOI = 0.05 for Caco2 cells) for 1 h at 37°C as described above, then the supernatants were harvested at 24 h postinfection and titrated by serial dilution in Vero-E6 cells. Viral titers are expressed as PFU/mL.

### Viral binding assay.

Cells were transfected with the indicated siRNA for 60 h. Then, the cells were transferred onto ice for 20 min. Then, 200 μL of ERA-EGFP (MOI = 10), HRB25 (MOI = 10), rVSV-SARS-CoV-S (MOI = 10), or rVSV-MERS-CoV-S (MOI = 10) was added to the cells at 4°C for 1 h. After being washed with pre-chilled PBS three times to remove the unbound virions, the cells and bound virions were lysed with TRIzol for qPCR to assess binding viral RNA.

### Viral internalization assay.

The internalization of HRB25, rVSV-SARS-CoV-S, or rVSV-MERS-CoV-S in Vero-E6 cells was performed as previously described (25). Briefly, cells were transfected with the indicated siRNA for 60 h and then transferred onto ice for 20 min. Then, 200 μL of ERA-EGFP (MOI = 10), HRB25 (MOI = 10), rVSV-SARS-CoV-S (MOI = 10), or rVSV-MERS-CoV-S (MOI = 10) was added to the cells at 4°C for 1 h. After removal of unbound virions by extensive washing with chilled PBS, the cells were moved to 37°C for 2.5 h (ERA-EGFP) or 1 h (HRB25, rVSV-SARS-CoV-S, and rVSV-MERS-CoV-S) to allow internalization. Then, the cells were washed for 3 min with trypsin or acid buffer/trypsin to remove the cell surface-bound viruses, and the cells were then lysed for total RNA extraction and subjected to qPCR to quantify the internalized viruses.

### qPCR.

To detect the TfR1 mRNA level and viral RNA level in cells, total RNA from cells was isolated using TRIzol reagent (Thermo Fisher); 2 μg of total RNA was used for reverse transcription (Vazyme). Relative mRNA expression was analyzed by using SYBR green qPCR Master Mix (Vazyme, Q711) with the indicated TfR1, RABV N, SARS-CoV-2 N, and VSV (Indiana strain) P gene-specific primers. The 2^-ΔΔCt^ method was used to calculate the relative gene expression level, with β-actin as the internal control (9). Experiments were done in 3 biological replicates. The qPCR primers used were as follows: TfR1 (human), forward (5′-CAGCCCAGCAGAAGCATT-3′) and reverse (5′-CCAAGAACCGCTTTATCCAG-3′); TfR1 (monkey), forward (5′-GGACCGTACTAAATTTCCCA-3′) and reverse (5′-ATCCAGGTGTGTAAGGGTCA-3′); RABV N gene, forward (5′-ATGAAGACTGTTCAGGACTGGTAT-3′) and reverse (5′-CCCTGGCTCAAACATTCTTCTTA-3′); SARS-CoV-2 N gene, forward (5′-GGGGAACTTCTCCTGCTAGAAT-3′) and reverse (5′-CAGACATTTTGCTCTCAAGCTG-3′); VSV (Indiana strain) P gene, forward (5′-GTGACGGACGAATGTCTCATAA-3′) and reverse (5′-TTTGACTCTCGCCTGATTGTAC-3′); β-actin (human), forward (5′-CGGGACCTGACTGACTACCTC-3′) and reverse (5′-CCATCTCTTGCTCGAAGTCCAG-3′); β-actin (mouse), forward (5′-CCTTCTTGGGTATGGAATCCTGTGG-3′) and reverse (5′-ACACAGAGTACTTGGGCTCAGGAGG-3′); β-actin (monkey), forward (5′-GACAGGATGCAGAAGGAGATTAC-3′) and reverse (5′-CTGCTTGCTGATCCACATCT-3′).

### Mass spectrometry.

To identify mGluR2-Flag-interacting proteins, four 15 cm dishes of HEK293 cells were transfected with mGluR2-Flag or pCAGGS-Flag plasmid for 48 h. The cells were then lysed with NP-40 lysis buffer (50 mM Tris [pH 7.4], 150 mM NaCl, 1% NP-40, 0.5% sodium deoxycholate) containing protease inhibitor for 1 h at 4°C with constant rotation. Cell lysates were centrifuged 12,000 rpm for 20 min at 4°C to remove cell debris. The soluble fraction was incubated for 4 h at 4°C with Protein G Agarose (Roche, 11243233001) to remove nonspecific binding proteins, followed by centrifugation at 5,000 *g* for 5 min at 4°C. The supernatant was mixed with anti-Flag antibody-conjugated agarose beads (Sigma, A2220) for 8 to 12 h at 4°C. The beads were washed six times with pre-chilled NP-40 lysis buffer, and then re-suspended in 50 μL of PBS buffer and mixed with protein sample loading buffer. After being boiled for 15 min, the samples were loaded onto a 4% to 12% gel for SDS-PAGE (Genscript), followed by mass spectrometry analysis. To improve accuracy, affinity purification coupled to mass spectrometry (AP-MS) was repeated three times independently for each bait protein.

Digestion of sample protein was performed according to the FASP procedure described previously (61). Briefly, protein bands on the SDS-PAGE gel were incubated with 200 μL of UA buffer (8 M Urea, 150 mM Tris-HCl pH 8.0) by repeated ultrafiltration (Microcon units, 10 kD) facilitated by centrifugation. Then, 100 μL of 0.05 M iodoacetamide in UA buffer was added to block reduced cysteine residues and the samples were incubated for 20 min in the dark. The filter was washed with 100 μL of UA buffer three times and then twice with 100 μL of 25 mM NH_4_HCO_3_. Finally, the gel pieces were digested with 3 μg of trypsin (Promega) in 40 μL of 25 mM NH_4_HCO_3_ at 37°C overnight and the resulting peptides were collected as the filtrate. The analysis experiments were performed on a Q Exactive mass spectrometer that was coupled to Easy nLC (Thermo Fisher Scientific). The peptide mixture was loaded onto a C18-reversed phase column (15 cm long, 75 μm inner diameter) packed in-house with RP-C18 5 μm resin in buffer A (0.1% Formic acid in HPLC-grade water) and separated with a linear gradient of buffer B (0.1% Formic acid in 84% acetonitrile) at a flow rate of 250 nl/min controlled by IntelliFlow technology over 60 min. Mass data were acquired using a data-dependent top 10 method dynamically choosing the most abundant precursor ions from the survey scan (300 to 1800 *m/z*) for HCD fragmentation. The resulting MS/MS data were processed using MaxQuant software version 1.3.0.5. Mass data were searched against Uniport Homo sapiens database (174046 total entries, downloaded 20190418). Trypsin/P was specified as the cleavage enzyme allowing up to 2 missing cleavages and a mass tolerance of 20 ppm for fragment ions. Carbamidomethyl on cysteines was specified as a fixed modification and oxidation on methionine was specified as a variable modification. Peptide confidence was set at high, and the peptide ion score was set ≥ 20. The cutoff of global false discovery rate (FDR) for peptide and protein identification was set to 0.01. The mass spectrometry proteomics data have been deposited to the ProteomeXchange Consortium (http://proteomecentral.proteomexchange.org) via the iProX partner repository (62) with the data set identifier PXD037306.

### Co-immunoprecipitation assay.

HEK293 cells were transfected with plasmids by using TransIT-293 transfection reagent according to the manufacturer’s instructions. Vero-E6 cells were transfected with plasmids by using ExFect transfection reagent (Vazyme, T101-AA) according to the manufacturer’s instructions. At 48 h posttransfection, cells were lysed with the gentle extraction buffer, NP-40 lysis buffer, or the rigorous extraction buffer, RIPA lysis buffer (50 mM Tris [pH 7.4], 150 mM NaCl, 1% Triton X-100, 1% sodium deoxycholate, 0.1% SDS) for 1 h at 4°C. Supernatant was collected and mixed with 40 μL of protein G agarose for 4 h at 4°C to remove nonspecific binding proteins in the supernatant. After being washed, the supernatant was mixed with anti-Flag antibody-conjugated agarose beads for 6 h at 4°C. The beads were isolated by centrifugation, washed five times with lysis buffer, and used for SDS-PAGE and Western blotting.

To detect the interaction between mGluR2-Flag and endogenous TfR1, HEK293 cells expressing mGluR2-Flag protein from two 15 cm dishes were used to extract cell plasma membrane by using the Minute Plasma Membrane Protein Isolation and Cell Fraction Kit (Invent Biotechnologies, SM-005). The yields were then used for the co-immunoprecipitation assay, SDS-PAGE, and Western blotting.

### Pulldown assay.

For the pulldown assay, mGluR2-GST (aa 19–567) and TfR1-GST (aa 89–760) proteins were expressed in E. coli and purified by FriendBio Technology. GST protein was used as the negative control. The purified GST-tagged proteins were respectively incubated with Glutathione Sepharose 4B beads (GE Healthcare Bio-science, 17-0756-01) at 4°C for 2 h. The beads were washed and incubated with the whole cell lysates from HEK293 cells expressing Flag-tagged or Myc-tagged proteins at 4°C for 5 h with constant rotation. After conjugation, the beads were washed five times with wash buffer (pH 8.5, 20 mM Tris, 500 mM NaCl, 2 mM EDTA) and re-suspended in PBS and protein sample loading buffer. The samples were then subjected to SDS-PAGE, and assessed by Western blotting.

### Western blot analysis.

Clarified cell lysate was diluted in denaturing SDS gel loading buffer, and boiled for 15 min. After denaturing, the samples were loaded onto a 4% to 12% gel for SDS-PAGE and separated by electrophoresis. Proteins were transferred to a polyvinylidene difluoride (PVDF) membrane (Merck Millipore, ISEQ00010). The PVDF membrane was blocked with 5% skim milk in PBS containing 0.1% Tween 20, and then incubated with the following primary antibodies: anti-Flag antibody (Genscript, A00187), anti-Myc antibody (Genscript, A00172), anti-GST antibody (Genscript, A00097), or anti-TfR1 antibody (Santa Cruz Biotechnology, sc-32272). Then, the PVDF membrane was washed three times with PBS and incubated with HRP-conjugated Goat anti-Mouse antibody (Genscript, A00160) and Goat anti-Rabbit antibody (Genscript, A00098). After three washes with PBST buffer, target protein bands were detected by using the enhanced chemiluminescence (ECL) reagent (Merck Millipore, WBLUR0500).

### Multiplex immunofluorescence.

N2a cells were cultured on Millicell EZ slide 4-Well Glass (Merck Millipore, PEZGS0416), and then infected with ERA-N-mCherry (MOI = 10) at 37°C for 10 min. Then, the cells were thoroughly washed with PBS and fixed with 3% paraformaldehyde. Multiplex immunofluorescence with Tyramide Signal Amplification (TSA) was performed by following a previously established protocol (25). Briefly, endogenous peroxidase activity was quenched. After permeabilization with 0.1% Triton X-100 and blocking steps (Zsbio, ZLI-9056), samples were incubated with primary antibodies, followed by HRP-conjugated secondary antibodies. Multiplex fluorescence labeling was performed using TSA-dendron-fluorophores (NEON 7-color Allround Discovery Kit for FFPE, Histova Biotechnology, NEFP750). A commercial antibody stripping buffer was employed to remove the primary and secondary antibodies while retaining the TSA signal by incubation for 30 min at 37°C. After a brief rinse, other antigens were serially detected by using spectrally different TSA reagents and following the above method. The primary antibodies used in this study were: TfR1 antibody (BD Pharmingen, 555534), mCherry antibody (Abcam, ab183628), mGluR2 antibody (Abcam, ab150387), and clathrin antibody (CST, 4796S). The secondary antibodies were: HRP-conjugated anti-rabbit IgG antibody (Zsbio, PV-6001), and HRP-conjugated anti-mouse IgG antibody (Zsbio, PV-6002). Images were acquired by using a Zeiss LSM880 laser-scanning confocal microscope equipped with Airyscan. The resolution of the acquired image was 1024 × 1024.

### Protein interaction competition assay.

ERA G-Myc, mGluR2-Flag, or pCAGGS-Flag were transfected into HEK293 cells, respectively. At 48 h posttransfection, cells were lysed with NP-40 buffer. The lysate was clarified and mixed with Protein G agarose. The supernatant containing mGluR2-Flag was mixed with anti-Flag antibody-conjugated agarose beads for 6 h at 4°C, then the beads were washed five times with NP-40 buffer. Increasing doses of TfR1-GST (0.5 μg, 1.5 μg, 2.5 μg) or GST (2.5 μg) were mixed with the aliquoted ERA G-Myc for 6 h at 4°C, respectively, then the mixture was added to the mGluR2-Flag-conjugated beads. After being incubated for 8 h at 4°C, the beads were washed five times with NP-40 buffer, and then re-suspended and subjected to SDS-PAGE and Western blotting. The protein interaction competition assays of TfR1-Flag and mGluR2-GST (1 μg, 3 μg), with SARS-CoV-2 S-Myc, SARS-CoV S-Myc, or MERS-CoV S-Myc were performed as described above.

### LY354740 agonist-triggered mGluR2 internalization assay.

For the LY354740 agonist activation assay, HEK293 cells or siRNA-transfected HEK293 cells were washed once with PBS, and then starved in PBS for 1 h at 37°C. The cells were then stimulated by incubation with 100 nM LY354740 in PBS for 30 min at 37°C. The cells were washed twice with PBS and fixed with 3% paraformaldehyde, and then the mGluR2 on the cell surface was stained with the mGluR2 antibody (Santa Cruz Biotechnology, sc-47135). The fluorescence intensity was quantified by using a Zeiss LSM880 laser-scanning confocal microscope.

### Statistical analysis.

Quantitative data are presented as means ± standard deviations (SDs) of 3 independent experiments or replicates. The statistical analysis of the normalized data was performed in Microsoft Excel using an unpaired two-tailed Student's *t* test. The statistical details are given in the respective figure legends. Significance levels were as follows: ns, not significant, *P < 0.05; **P < 0.01; and ***P < 0.001.

The co-localization analysis of RABV, mGluR2, TfR1, and clathrin was performed as follows: All channels were processed by using the “surface module”. The surface results of the red channel (RABV) and purple channel (mGluR2) were merge into one channel by using the “coloc module”. The new channel was processed by using the “spot module”. The spots from the new channel were inputted as “vesicle,” and the surface results of the yellow channel (clathrin) and green channel (TfR1) were inputted as “cell” and “nuclei,” respectively, under the “cell module”. Spots that colocalized with the yellow and green channels (displayed as arrows in the rendered image) were counted.
